# Current Options for Determining Fracture Union

**DOI:** 10.1155/2014/708574

**Published:** 2014-09-14

**Authors:** Saam Morshed

**Affiliations:** Department of Orthopaedic Surgery, University of San Francisco School of Medicine, San Francisco, CA 94143-0410, USA

## Abstract

Determining whether a bone fracture is healed is one of the most important and fundamental clinical determinations made in orthopaedics. However, there are currently no standardized methods of assessing fracture union, which in turn has created significant disagreement among orthopaedic surgeons in both clinical and research settings. An extensive amount of research has been dedicated to finding novel and reliable ways of determining healing with some promising results. Recent advancements in imaging techniques and introduction of new radiographic scores have helped decrease the amount of disagreement on this topic among physicians. The knowledge gained from biomechanical studies of bone healing has helped us refine our tools and create more efficient and practical research instruments. Additionally, a deeper understanding of the molecular pathways involved in the bone healing process has led to emergence of serologic markers as possible candidates in assessment of fracture union. In addition to our current physician centered methods, patient-centered approaches assessing quality of life and function are gaining popularity in assessment of fracture union. Despite these advances, assessment of union remains an imperfect practice in the clinical setting. Therefore, clinicians need to draw on multiple modalities that directly and indirectly measure or correlate with bone healing when counseling patients.

## 1. Introduction

There are about 6 million fractures in the United States annually and 5–10% of these fractures proceed to nonunion [1]. The risk of nonunion is increased based on certain patient factors such as smoking habit or diabetes and varies by location of fracture with those of the scaphoid waist, neck of femur, and open fractures of the tibia being especially susceptible [2–6]. Nonunions are associated with significantly higher rate of healthcare resource use, drastically higher per patient cost, and use of stronger opioid medications [7–10]. Infection can present as a delay or failure of fracture repair, and the clinician should always consider this in the differential diagnosis. Determining when a fracture is healed is a routine part of orthopaedic clinical care. It is crucial to making the right clinical decision for patients including determining their weight-bearing status, appropriate time for hardware removal, and diagnosis and treatment of nonunions. It is also tremendously important in interpreting research studies on treatment and therapeutics of fracture repair. Therefore, a valid and standard definition of fracture union should be an essential and fundamental goal in orthopaedics. Lack of such standardized and unified definition can lead to questionable and controversial results or more importantly expose the patients to additional and avoidable risks. The recent controversies surrounding the use of recombinant human bone morphogenetic protein-2 are an example of how assessment of fracture union is crucial to generating valid inferences in pivotal studies leading to approval of novel therapeutic modalities [11–13].

There has been considerable clinical and basic science research dedicated to better defining fracture healing and developing more efficient diagnostic tools for earlier and more accurate diagnosis of nonunions. However, it still largely remains a subjective topic and there is a significant amount of disagreement among physicians in regard to when a fracture is healed. A survey of 444 orthopaedic surgeons a decade ago identified that there is a lack of consensus in defining delayed union and nonunion in tibial fractures among orthopaedic surgeons. There was considerable disagreement in both clinical and radiographic criteria to define fracture union as well as the average time required for diagnosis of delayed or failed union [14]. The same level of disagreement and variability seems to exist among researchers in regard to clinical and radiographic definitions of fracture healing. A systematic review of 92 studies published between year 2000 and 2006 showed similar trends of a lack of objective tools to radiographically or clinically assess fracture healing [15].

This subjectivity and lack of agreement among clinicians and researchers in definition of fracture union are a major obstacle in conducting clinical trials in this field. Recently, there has been some effort to create standardized fracture union checklists. Though not completely validated, the initial assessments of these diagnostic tools are promising. The goal of this paper is to review the current options for determining fracture union and to explore recent advancements made in the field. The target audience of this review is the clinician who takes care of patients with bone fracture, be they a primary care physician or orthopaedic surgeon, as the diagnosis of fracture healing is fundamental to caring for these patients at any level of specialization.

## 2. Biology of Fracture Healing

To fully grasp the reason behind the lack of a gold standard in determining union, it is important to understand the complex molecular pathways and mechanical factors involved in bone healing. A detailed discussion of the molecules and cytokines involved in bone healing is beyond the scope of our review; however, a large number of these factors have been identified and extensively studied in both animal and human models [17–19]. Skeletal tissue has a great regenerative ability and it is now known that bone is one of the few tissues that can heal without forming fibrous scar tissue and regain its prefracture mechanical properties. A significant number of factors work in a highly coordinated and complex fashion at the molecular level to achieve this goal.

It is helpful to think of the bone healing process in a stepwise fashion, even though in reality there is a great overlap among these different stages. In general, it is possible to divide this process into an initial hematoma formation step, followed by inflammation, proliferation and differentiation, and eventually ossification and remodeling [20]. Shortly after a fracture ocurrs, the vascular injury to periosteum, endosteum, and the surrounding soft tissue causes hypoperfusion in the adjacent area. The coagulation cascade is activated which leads to the formation of a hematoma rich in platelets and macrophages. Cytokines from these macrophages initiate an inflammatory response, including increased blood flow and vascular permeability at the fracture site. Mechanical and molecular signals dictate what happens subsequently. Fracture healing can occur either through direct intramembranous healing or more commonly through indirect or secondary healing. The major difference between these two pathways is that direct healing requires absolute stability and lack of interfragmentary motion, whereas, in secondary healing, presence of interfragmentary motion at the site of fracture creates relative stability. In secondary healing, this mechanical stimulation in addition to the activity of the inflammatory molecules leads to formation of fracture callus followed by woven bone which is eventually remodeled to lamellar bone.

At a molecular level secretion of numerous cytokines and proinflammatory factors coordinate these complex pathways. Tumor necrosis factor-*α* (TNF-*α*), interleukin-1 (IL-1), IL-6, IL-11, and IL-18 are responsible for the initial inflammatory response [21]. Mesenchymal stem cells are recruited from the surrounding soft tissue and differentiate into osteogenic cells which are involved in generation of cartilaginous and periosteal bony callus [22]. Revascularization, an essential component of bone healing, is achieved through different molecular pathways requiring either angiopoietin or vascular endothelial growth factors (VEGF) [23]. VEGF's importance in the process of bone repair has been shown in a number of studies involving animal models [24, 25]. As the collagen matrix is invaded by blood vessels, the mineralization of the soft callus occurs through the activity of osteoblasts resulting in hard callus, which is remodeled into lamellar bone. Inhibition of angiogenesis in rats with closed femoral fractures completely prevented healing and resulted in atrophic nonunions [26]. On the other hand, inadequate fixation in presence of good vascularity has been shown to lead to hypertrophic nonunion [22, 27]. Therefore, successful fracture healing requires a balanced interaction between biological and biomechanical forces.

As evident, fracture healing is a continuous and complex biological and molecular process. However, in the clinical setting, physicians often dichotomize healing to aid in clinical decision-making and draw conclusions about efficacy of treatment. This oversimplification can lead to loss of valuable information along the spectrum of healing and more importantly misdiagnosis and misguided treatment decisions.

## 3. Nonunion

**Figure 1:**
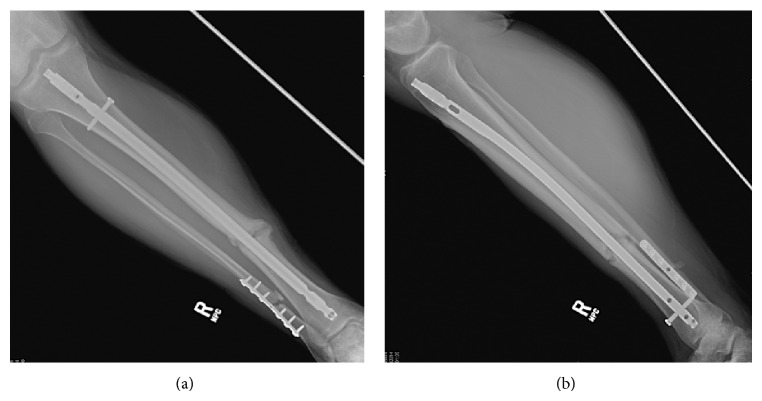
((a) and (b)) Radiographs of a hypertrophic tibial nonunion in a 35-year-old man ten months status after medullary fixation of an open tibial shaft fracture with persistent pain and inability to weight bare. Note abundant callus formation but persistent fracture line.

**Figure 2:**
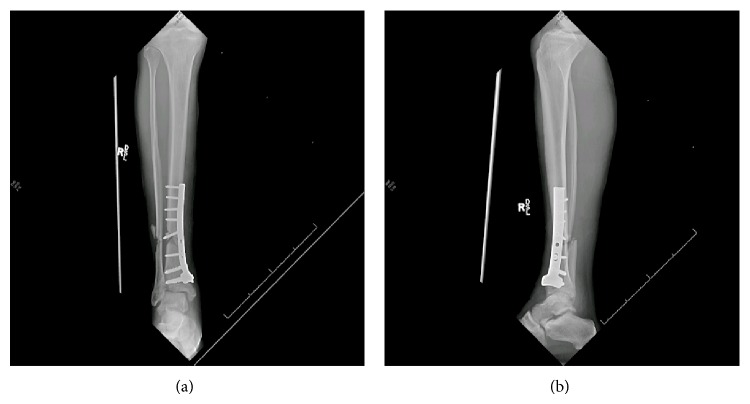
((a) and (b)) Radiographs of an atrophic nonunion of the tibia with hardware failure one year after motorcycle collision resulting in an open tibial fracture. In this patient, little or no callus is evident.

There is currently no accepted standardized definition of fracture nonunion among orthopaedic surgeons. According to the definition provided by the American Food and Drug Administration (FDA) a minimum of at least nine months has to elapse since the initial injury and there should be no signs of healing for the final three months for diagnosis of fracture nonunion [28]. There are a few different classification systems of nonunions, but nonunions are most commonly divided into two categories of hypervascular nonunion and avascular nonunion [29, 30]. In hypervascular nonunions, also known as hypertrophic nonunion, fracture ends are vascular and are capable of biological activity. There is evidence of callus formation around the fracture site (Figure 1) and it is thought to be in response to excessive micromotion at the fracture site [31]. Avascular nonunions, also known as atrophic nonunion, are caused by avascularity or poor blood supply of the fracture ends [32, 33]. There is no or minimal callus formation and fracture line remains visible (Figure 2). This type of nonunion requires biological enhancement in addition to adequate immobilization to heal [29].

## 4. Measures of Healing

Our current available tools in assessment of fracture healing can be broadly divided into four categories: (1) imaging studies, (2) mechanical assessment, (3) serologic markers, and (4) clinical examination. We will explore each of these categories and their current use in clinical or research settings in detail.

### 4.1. Imaging Measures

Despite their limitations, radiographic assessment has remained a crucial tool in determining fracture healing. This stems from clinicians' familiarity with plain radiography and their widespread availability and accessibility. Bhandari et al. showed in an international survey of 444 orthopaedic surgeons in 2002 that 39.7% to 45.8% of surgeons always used radiographic data, including callus size, cortical continuity, and progressive loss of fracture line in assessment of tibial fracture healing [14]. Despite developments of advanced imaging techniques to quantitatively and qualitatively assess bone health and fracture healing, plain radiography remains the most commonly used radiographic tool for this purpose. This is due to lower cost, wider availability, and lower radiation exposure of plain radiography compared to other available modalities. However, the few studies that looked at reliability of plain radiography in detecting fracture healing concluded that radiographs do not define union with enough accuracy and are generally inconclusive in determining the stage of union [34–36]. Research into validation and standardization of these radiographic tools is surprisingly sparse. There have been a few recent studies that attempted to standardize radiographic healing criteria for tibia and femur fractures with promising initial results [16, 37–39]. We review these studies along with a few other imaging modalities used in determination of union, including computed tomography and ultrasound.

#### 4.1.1. Radiographic Union Scores

The teams at the University of Toronto and McMaster University have recently developed two radiographic scoring systems, radiographic union score for hip (RUSH) and radiographic union score for tibia (RUST), that have been shown to increase agreement among surgeons and radiologists in assessing fracture repair [16, 38–40]. After pointing out the limitations of older radiographic scoring systems, they showed that assessment of the number of cortices bridged by callus had higher reliability in determining healing [41]. Based on this finding they attempted to improve accuracy of radiographic fracture union assessment by developing scaling systems that were mainly based on the appearance of the cortex on plain films.

**Figure 3:**
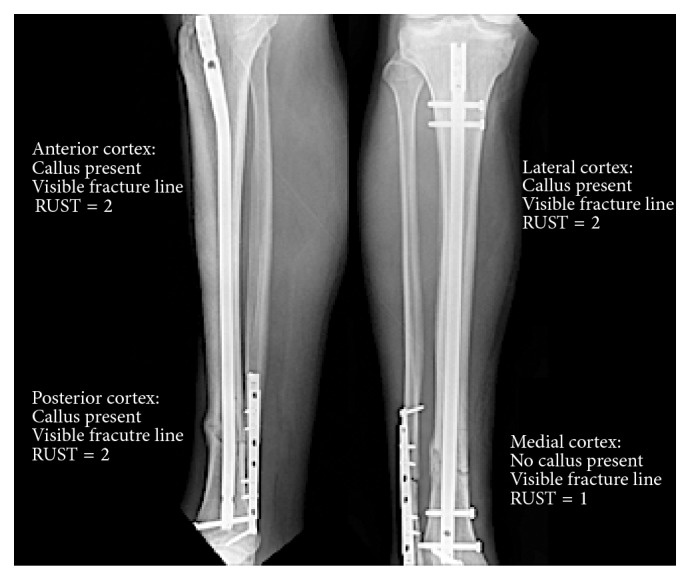
Assignment of the RUST in a patient with distal tibial shaft fracture at 3 months postoperatively. Overall RUST = 7.

The RUST is based on callus formation and visibility of fracture line at 4 cortices observed on AP and lateral radiographs (Figure 3). Minimum score of 4 indicates no healing and maximum of 12 indicates a healed fracture. Score for each cortex is assigned according to the criteria shown in Table 1. Whelan et al. looked at 45 sets of anteroposterior and lateral radiographs of tibial shaft fractures treated with intramedullary nails [39]. Seven reviewers, including orthopaedic residents, orthopaedic surgeons, and orthopaedic traumatologists independently evaluated the images for fracture healing using the RUST score. Agreement was measured using intraclass correlation coefficients (ICC) with 95% confidence intervals (CI). They found that overall interobserver agreement was substantial at both the initial assessment (ICC = 0.86, 95% CI 0.79–0.91) and 9 weeks after (ICC = 0.88, 95% CI 0.80–0.96). However, since there is currently no gold standard to compare the RUST to, they concluded that further research is required to fully validate this scoring system as a clinical tool.

**Figure 4:**
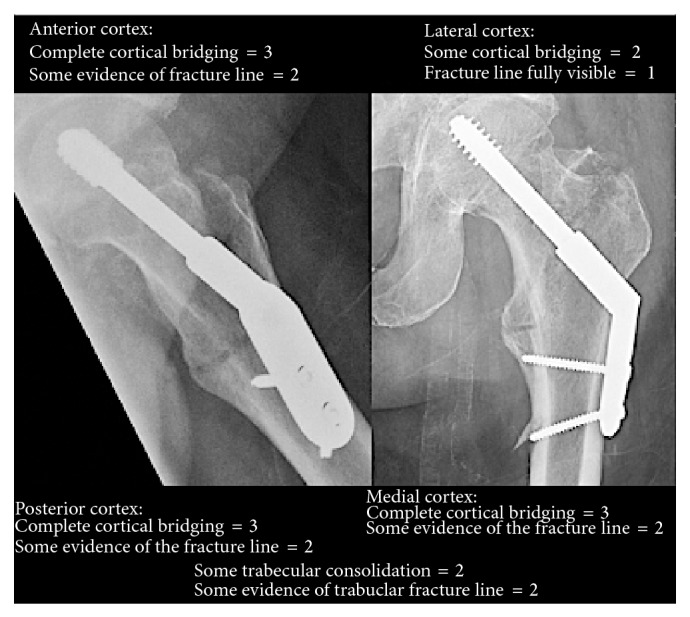
Assignment of the RUSH in a patient with an acute intertrochanteric fracture at 6 weeks postoperatively. The overall score in this patient is 22. As evident, the RUSH checklist incorporates cortical and trabecular bridging and fracture line disappearance in its scoring system.

Similarly, the RUSH provides a standardized radiographic assessment of hip fracture healing based on absence or presence of bridging and appearance of the fracture line.  Figure 4 provides an example of using the RUSH in assessment of fracture union.

Bhandari et al. reviewed 150 cases of femoral neck fractures at two time points by a panel of three radiologists and three orthopaedic surgeons [37]. Reviewers were blinded to the time that the images were taken postoperatively. They reviewed each image to subjectively determine healing using anteroposterior and lateral images followed by assessment of the same images using the RUSH. They found higher agreement of fracture healing with use of the RUSH (ICC = 0.53, 95% CI: 0.30–0.69) compared to subjective assessment (ICC = 0.22, CI: 0.01–0.41). The same group conducted another similar study in which the six reviewers (three orthopaedic surgeons and three radiologists) had access to the time the images were taken after injury [38]. They assessed fracture healing using sequential radiographs in 100 patients with femoral neck fractures and 100 patients with intertrochanteric fractures. Agreement was almost perfect for both femoral neck and intertrochanteric fractures using RUSH score (ICC = 0.85, 95% CI: 0.82–0.87, and ICC = 0.88, 95% CI: 0.86–0.90, resp.) The RUSH score could potentially be used as a clinical tool given the evidence of increased reliability and agreement among clinicians. However, both the RUST and RUSH currently remain to be validated in terms of prediction of fracture union. This requires larger clinical studies to compare data from the RUST and RUSH with other available outcome measures of healing including physical exam findings, other imaging modalities, and biomechanical data.

#### 4.1.2. Computed Tomography

**Figure 5:**
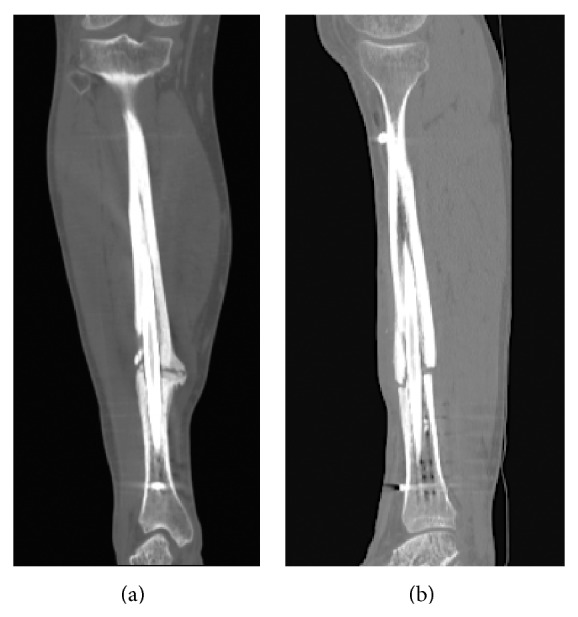


Computed tomography (CT) is superior to plain radiography in assessment of union and visualizing of fracture in presence of abundant callus or overlaying cast (Figure 5) [42]. There have been studies to test accuracy and efficacy of computed tomography in assessment of fracture union in clinical settings. Bhattacharyya et al. showed that computed tomography has 100% sensitivity for detecting nonunion; however, it is limited by a low specificity of 62% [43]. Three of the 35 patients in the study were misdiagnosed as tibial nonunion based on CT scan findings but were actually healed when fracture was visualized during surgical intervention. In a study of 18 patients with complex fractures of tibia shaft stabilized initially with external fixator, it was shown that an increase of more than 50% callus formation after 12 weeks on CT was an indicator of stability with sensitivity of 100% and specificity of 83% [44]. These findings correlated well with the data obtained from refractometry, a noninvasive method of measuring stability in fractures treated with external fixators. In another study investigators compared quantitative and qualitative changes of fracture healing in 39 patients with closed fractures of distal radius, tibia and/or fibular malleoli, or tibial shaft using both computed tomography and conventional radiography [45]. They found that early manifestations of healing, including blurring of fracture margins and formation of external callus, were observed earlier with CT scan. Most of the discrepancies between X-ray and CT scan findings were in periarticular and metaphyseal injuries. Overall, the findings of both modalities matched in 64% of cases. Overall the investigators concluded that CT scans have some advantages over radiographs in early detection of fracture healing in radius fractures. A limitation of CT is beam-hardening artifact from internal and external fixation. Despite reductions in image degradation from these artifacts using modern software, resolution is still affected when the region of interest is adjacent to metal implants. Currently, cost and radiation dose of CT scans limit their widespread use as the main clinical assessment tool for assessment of fracture healing despite evidence of their good diagnostic accuracy and correlation with other clinical markers of healing.

A new technology called virtual stress testing (VST) draws on improving resolution provided by CT-based finite element analysis. Finite element analysis (FE) is a mathematical tool initially designed for structural and stress analysis of buildings, bridges, and other architectural structures. Its use in orthopaedics involves simulation of either static problems, such as weight bearing capacity of implants and prosthetics or dynamic problems, such as fall analysis [46]. Finite element analysis uses information from CT images to provide quantitative assessment of bone strength [47]. Orwoll and his colleagues recently showed that biomechanical data obtained from finite element analysis correlate well with risk of hip fractures in men above the age of 65 [47]. This correlation remained statistically significant after adjusting for age and BMI.

Recently this technology was expanded from prediction of fracture risk to evaluation of fracture repair. In a pilot study Petfield used VST in complex tibia fractures treated with ring fixators to identify patients who would have a clinical event including refracture, malunion, or need for surgical revision if their hardware was removed. They retrospectively included 66 patients with CT scans of their fracture 2–4 weeks prior to removal of their ring fixators. With virtual stress testing they were able to use the information obtained from CT images and simulate multiple loading conditions after subtracting the mechanical contribution of the external fixator and therefore predict outcomes like axial compression, bending, and area of tissue failure. Eleven patients eventually had one of the above clinical events. Using quantitative data on failed tissue percentage and bone strength to body weight ratio, they were able to predict 9 of these 11 events [48]. More prospective studies with large sample sizes are required at this point to validate this technology and expand its use to other forms of internal fixations.

#### 4.1.3. Ultrasound

Ultrasound is unable to penetrate cortical bone, but there is evidence that it is able to detect callus formation before radiographic changes are visible [49, 50]. Following the promising results of their pilot study in which ultrasound was able to correctly predict union at a much shorter period of time compared to X-ray [51], Moed conducted a larger prospective study which showed that ultrasound findings at 6 and 9 weeks have a 97% positive predictive value (95% CI: 0.9-1) and 100% sensitivity in determining fracture healing in patients with acute tibial fractures treated with locked intramedullary nailing [52]. Time to determination of healing was also shorter using ultrasound (6.5 weeks) compared to nineteen-week average of radiographic data (*P* < 0.001). Ultrasound has additional advantages over other imaging modalities including lower cost, no ionizing radiation exposure, and being noninvasive. However its use and interpretation of findings are thought to be highly dependent on operator's expertise. Furthermore, thick layers of soft tissue can obscure adequate view of bones with ultrasound. As ultrasound technology advances, many of these limitations will likely be addressed. As with other imaging modalities, further prospective validation is required.

#### 4.1.4. Positron Emission Tomography

Positron emission tomography (PET) imaging generates imaging based on metabolic activity of different tissues. It has been historically used in detection of highly metabolic active tumors. A study in 2007 used PET scan with ^18^F-fluoride ion in assessment of bone healing in rats with femur fractures [53]. ^18^F-fluoride ion deposits in regions of the bone with high osteoblastic activity and high rate of turnover, such as endosteal and periosteal surfaces [54, 55]. In this study, one group of rats received intramedullary fixation for their femur fractures while in the second group investigators placed spacers at fracture sites to interfere with the healing process throughout the study. They evaluated the bone healing of both groups by weekly PET scans and plain radiographs. In treatment group uptake of ^18^F-fluoride ion increased consistently between 1–3 weeks and remained elevated at 4 weeks after treatment. Radiographic and histologic analysis of femurs in this group also showed clear signs of healing. In contrast, ^18^F-fluoride ion uptake in the group of rats with spacers was significantly lower at all time points throughout the study compared to the treatment group (*P* < 0.005). They concluded that ^18^F-fluoride ion PET could potentially play an important part in assessment of fracture healing given its ability to quantitatively monitor metabolic activity and provide objective evaluation of fracture repair.

### 4.2. Mechanical Property Testing

Mechanical testing measures fracture stiffness and stability. Modulating stability is a concept that orthopaedic surgeons think about and deal with on a daily basis. Increase in fracture stiffness is an indication of healing and it also correlates well with strength in the early phases of callus formation after injury [56, 57]. Biomechanical testing and vibrational analysis both utilize this concept in assessment of fracture healing. While the majority of these modalities cannot assist in assessment of fractures treated with internal fixation, many are still in use as research tools and may have some clinical role when external fixation is used.

#### 4.2.1. Biomechanical Testing

Biomechanical testing methods can be divided into direct and indirect measurement of stiffness. In direct measurement displacement angle across the fracture is measured by radiograph or surface measurements using four-point bending in the setting of applied load [58, 59]. The degree of deflection by the bending moment was assumed to be inversely proportional to the stability of the fracture union. The authors referred to this technique as “shift comparison” and introduced it as a quantitative method of measuring stability. This technique requires that no cast or hardware be present. Marsh defined nonunion in the study of 43 isolated closed tibial shaft fractures as failure to reach a stiffness of 7 Nm per degree by 20 weeks after injury since none of the fractures that reached this value failed to heal [60]. There was also high degree of correlation between stiffness measurements with injury severity and functional outcomes (SF-36) at 6 months. He explained delayed union as the cessation of periosteal activity before the completion of fracture bridging and nonunion as the cessation of both periosteal and endosteal responses with no bridging in the case of conservatively managed fractures.

In indirect testing fracture stiffness is measured by using strain-gauge units attached to external fixators to measure the strain in the fixator column [57]. Jorgensen measured fracture bending at a known amount of load in tibial fractures [61]. Richardson et al. noted that this method provides only indirect measurement of changes in stress in the fixator as the fracture heals; however, there are currently methods available to measure absolute values of stiffness using the same system [57, 62, 63]. Using this technique, Richardson et al. showed that most patients were able to weight-bear without support when their fracture stiffness reached 15 Nm per degree and that use of this threshold as compared to clinical and radiographic assessment was a better predictor of likelihood of refracture (*P* = 0.02) and also decreased the time to independent weight-bearing (*P* = 0.02) [57, 64].

#### 4.2.2. Vibrational Analysis

Vibrational testing uses either resonant frequency or computerized sonometry to assess mechanical properties of healing bones. The advantage of these methods compared to biomechanical testing is that they are noninvasive and painless. Resonant frequency analysis (RFA) is based on the principle that there is a direct correlation between the natural frequency of a beam and its stiffness. Long bones act as beams and therefore the same principle can be applied to long bones [65]. Early studies were done by Jurist who proposed that estimation of Young's modulus of bones* in vivo *could be used to assess bone quality [66]. His lab measured resonant frequency by detecting bones' response to vibratory changes. Biological and physical changes in bone throughout the healing process change this resonant frequency [65–68]. Benirschke et al. showed that resonant frequency correlated well with both bending rigidity (*r* ^2^ = 0.815) of tibia and time to fracture healing [67]. Lowet used finite element modeling to show that there is a linear correlation between resonant frequencies and torsional stiffness of the healing tibia once callus stiffness reached 5% or higher of the stiffness of the intact bone [69]. Despite all these evidences for resonant frequency analysis as a quantitative tool for assessment of fracture healing, there are shortcomings associated with this method that limit its use. In a study of 74 tibial fractures it was shown that resonant frequency analysis was significantly inaccurate in assessment of healing in fractures of the proximal fourth of the tibia and fractures treated with interlocking nails [65]. They detected similar errors in a few patients treated with external fixators. Many of these fractures were falsely identified as healed by resonant frequency analysis. Authors explained this by proposing that RFA was most probably measuring the stiffness of the fixation instead of the healing fracture. Also quantity and quality of the overlaying soft tissue have a significant impact on the measurements of vibrational testing [70].

Quantitative ultrasonometry has also been studied for assessment of its efficacy in measurement of bone healing. Early studies in animal models demonstrated that ultrasound propagation velocity (USPV) across fractures approaches values of normal bone throughout the healing process [71]. Fellinger et al. used a system consisting of two sound transducers on two ends of tibial fractures with external fixators to evaluate the healing process by detecting sound transmission across these fractures [72]. Using computerized analysis of vibration reaction and sound propagation along fractures they were able to detect early signs of delayed union before radiographic signs were evident. Since then more precise devices have been developed and tested* in vitro* with similar results, showing high accuracy in predicting simulated fracture gap using ultrasound propagation velocity (*r* ^2^ = 0.994) [73]. Investigators used a tibia phantom with simulated transverse fractures for this study, which as they acknowledged is a simplification of actual clinical fractures with various anatomical characteristics and amount of soft tissue damage. Overlaying soft tissues limit* in vivo* studies of computerized sonometry to subcutaneous bones. Lack of large-scale reports of diagnostic accuracy and reliability of this modality in assessment of healing of various types of fractures in humans is the major barrier to its transition into the clinical and research practice.

## 5. Serologic Markers

As discussed above, prediction and early detection of nonunions could lead to lower medical costs and better clinical outcomes for patients. Given what we know about the early local and systemic molecular changes following a fracture, serologic biomarkers are gaining popularity as possible early predictors of fracture healing [74–77]. While C-reactive protein (CRP) and erythrocyte sedimentation rate (ESR) are commonly used in the evaluation of nonunion, particularly those where infection is suspected, much research has recently been focused on the identification of more sensitive and specific markers of delayed or failed fracture repair. Studies in human and animal models have identified many candidate biomarkers and potential limitations associated with their use as clinical diagnostic tools.

Moghaddam et al. conducted a prospective cohort study to assess changes in serum concentrations of a few serologic markers in normal and delayed fracture healing [78]. He was able to show significantly lower levels of tartrate-resistant acid phosphatase 5b (TRACP 5b) and C-terminal cross-linking telopeptide of type I collagen (CTX) in patients who developed nonunions compared to patients with normal healing. TRACP 5b is a direct marker of osteoclastic activity and bone resorption, while CTX is an indirect measure of osteoclastic activity by reflecting collagen degradation. Following a bone fracture, increased bone metabolism is observed in patients with normal healing. This is reflected by a sudden increase in osteoclastic markers, namely, CTX and TRACP 5b, during the first few weeks [79]. However, this increase was not observed in the delayed healing group, indicating a lower initial bone turnover following their injury. On the other hand, osteoblastic markers, total N-terminal propeptide of type I collagen (PINP), and bone specific alkaline phosphatase (BAP) initially decrease followed by an increase in both treatment and control groups without any significant difference between the two groups.

Another serologic marker of healing that has been extensively studied within the past decade is transforming growth factor-beta 1 (TGF-*β*1) [80–84]. It is a member of the TGF-*β* family and has been shown to be an essential regulatory molecule in fracture healing. It has been detected in callus of human and animal fracture models [74, 85] and its systemic and local administration enhanced bone remodeling and fracture healing in animal models [82, 86]. Zimmermann et al. prospectively assessed systemic changes of TGF-*β*1 levels in patients with delayed healing and nonunion of long bone fractures [87]. He found that in both normal and nonhealing groups serum level of TGF-*β*1 increased within the first 2 weeks after fracture. However, the delayed healing group had a faster decline of serum concentration between 2 and 4 weeks after trauma and its level was significantly lower in the delayed fracture-healing group at 4 weeks. However, a more recent study by Sarahrudi found no significant differences in the TGF-*β*1 concentrations of delayed and normal fracture healing groups [88].

Collagen III amino-terminal propeptide (PIIINP) is the N-terminal peptide cleaved from type-III procollagen during the process of type-III collagen synthesis [75]. Stoffel et al. [89] showed PIIINP becomes elevated during fracture healing and reaches its maximum at two weeks in malleolar fractures and 12 weeks in tibial fractures. Its level decreases afterwards and normalizes, which preceded radiographic and clinical evidence of healing. Kurdy [90] showed in 20 patients with isolated tibial shaft fractures that serum PIIINP levels were significantly higher in the nonhealing group at 10 weeks. This difference in PIIINP levels between healing and nonhealing groups was evident within the first 10 weeks after the initial injury.

Despite these encouraging findings there are a few issues that make the use of these biomarkers as diagnostic tools problematic. Secretion of many of the cytokines and biologic markers is also influenced by other factors. For example, systemic levels of TGF-*β* were found to vary based on smoking status, age, gender, diabetes mellitus, and chronic alcohol abuse at different time points [91]. The same factors, excluding alcohol abuse, have been shown to affect expression of macrophage colony stimulating factor (M-CSF) and vascular endothelial growth factors (VEGF) [92]. A recent systematic review concluded after thorough analysis of data presented in 44 studies that no recommendations in terms of clinical use of these serologic biomarkers can be made at this point [93]. Some of the limitations they identified include the small number of patients recruited in these studies, genetic heterogeneity among individuals, variation of the populations used in studies, and lack of large randomized trials in assessment of nonunion biomarkers.

## 6. Clinical Assessment of Healing

Despite all the advancements in developing fracture assessment instruments reviewed above, physical exam remains one of the mainstays of determining fracture union in the clinical setting. Patients with suspected nonunion should always be inspected for local signs of infection such as erythema, drainage, and wound problems. In a recent international survey of 335 orthopaedic surgeons, 88% of the participants agreed that radiographic and clinical data are required for adequate definition of union [94]. For delayed union and nonunion a majority of the respondents (83% and 84%, resp.) indicated that lack of weight-bearing ability was the most important clinical criterion for diagnosis, followed by fracture pain (78% and 74%, resp.) and weight-bearing status (48% and 51%, resp.). In a systematic review in 2008, out of fifty nine studies that used clinical criteria in defining union, absence of pain or tenderness at the fracture site on weight-bearing (31/59), absence of pain on palpation at the site of fracture (23/59), and the ability to weight-bear (12/59) were the most commonly used criteria to define fracture healing [15]. Lack of a standardized clinical definition of union contributes to this observed variability among clinicians and researchers in defining union.

Weight-bearing status has been shown to correlate relatively well with fracture stiffness in tibial fractures treated with external fixation [95]; however, physicians' ability to judge stiffness and weight-bearing ability based on physical exam alone is not very reliable. Webb et al. showed that manual assessment of stiffness by orthopaedic surgeons was not superior to that of medical students [96]. Additionally, it was shown that physicians, regardless of number of years of experience, are not reliable in judging stability with increasing stiffness of fractures [97]. As discussed above, other more reliable biomechanical modalities of assessment of stiffness are not currently used as clinical tools. Pain on palpation at the site of injury is also currently widely used among physicians to judge union; however, it is a highly subjective outcome given individual and cultural differences in perception and tolerance level of pain among the population.

**Figure 6:**
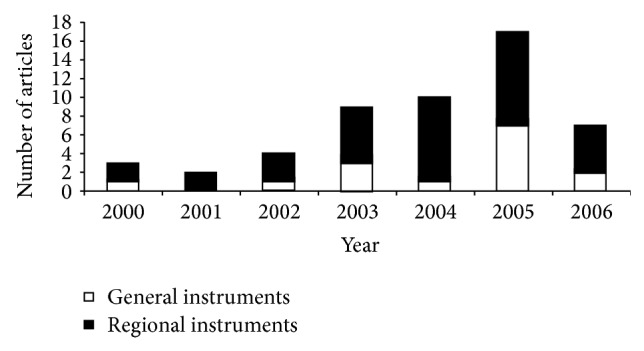
Distribution of general and region-specific instrument usage over time from 2000 to 2006 showing an increase in use of regional questionnaires between 2000 and 2005.

It is important to consider that patients might think very differently about the process of healing compared to physicians and other healthcare professionals. None of the tools we described so far assesses patients' goals and expectations in terms of their daily physical and mental health during the healing period. Therefore, use of tools to evaluate patient-reported outcome measures should be an important part of both research and clinical assessment of fracture healing. The increase in number of clinical studies between 2000 and 2005 that used patient-reported health-related questionnaires in assessment of fracture healing could indicate a shift towards a more patient-centered approach in dealing with this topic (Figure 6) [15].

The currently available patient-reported functional outcome assessment tools either measure general physical and psychological health, as in the Short Form-36 (SF-36) [98, 99], or are disease-specific, as in disability of the arm, shoulder, and hand (DASH) [100] or Western Ontario McMaster Arthritis Index (WOMAC) [101]. Disease-specific or region-specific questionnaires generally provide information on pain, physical status, and functional assessment of a specific body region, whereas general health questionnaires like SF-36 are generic measures of functional wellbeing and mental health [102]. Another class of questionnaires is health related quality of life (HRQoL) that measures patients' quality of life and how it is affected by a disease, disability, or treatment. Health Utility Index [103] and EuroQol-5D [104] are examples of the health related quality of life questionnaires. In the future, computer-assisted testing implementing item response theory is likely to streamline the process of gathering patient reported outcomes as evidenced by the National Institutes of Health PROMISE initiative [105]. These more efficient instruments are currently being validated in a number of different orthopaedic clinical settings including orthopaedic trauma.

## 7. Conclusion

As evident by now, fracture healing requires a complex interplay of biological pathways and mechanical forces. This process occurs in a continuum that varies dramatically based on fracture location, type, choice of treatment, and other host and injury related factors. Therefore, dichotomization of this complicated phenomenon is a clear oversimplification with subsequent loss of valuable information. This lack of standardized definition of fracture healing impairs our ability to compare findings from various studies on this topic. Recent developments of the RUSH and RUST score aim to improve reliability among assessors. Serologic markers also show promising results in more accurately predicting rate and quality of fracture healing; however, genetic and environmental variations among individuals limit their current clinical utility.

The future direction of fracture healing assessment should focus on further validation of the current available tools and development of better physician-assessed and patient-assessed instruments in measurement of union. The quality of these tools should be determined by evaluating their measurement properties, including reliability, validity, and reproducibility of results [106]. Defining a gold standard that incorporates all the different clinical, radiographic, biological, and biomechanical factors of healing has proven to be a difficult task. An endpoint Adjudication Committee can and should help increase agreement in assessment of fracture healing in clinical trials [107], as long as it is recognized that this may incompletely measure the impact of a treatment on overall injury recovery and health-related quality of life. Measurement of patient-reported outcomes can enhance our understanding of what radiographs and physical examination tell us about the degree of bone healing, though they may also enlighten clinicians and researchers as to how little an intervention meant to affect fracture repair impacts general health.

## Figures and Tables

**Table 1:** Individual cortex score based on radiographic findings. These scores are added to calculate the RUST [16].

Score per cortex	Callus	Fracture line
1	Absent	Visible
2	Present	Visible
3	Present	Invisible
